# Notes of an Anomalous Case of Traumatic Tetanus, Which Terminated Favourably

**Published:** 1845-06

**Authors:** Edward R. Squibb


*Notes of an Anomalous Case of Traumatic Tetanus, which terminated favourably.* By Edward R. Squibb, M. D.

The following case was treated under the direction and superintendance of Professor Franklin Bache, to whom the writer is indebted for much valuable instruction, received at his hands. It occurred during the Professor's term of service in the Dispensary of the Jefferson Medical College, and possesses some interest, from its anomalous character and fortunate issue.

Joseph J., æt. 20, a short, thick set, muscular man, with brown hair, hazel eyes, and a florid, although not very full, countenance, with firm and powerful muscles, and the brawny appearance of full health, which he has always enjoyed, although much exposed to cold and damp by his occupation,—that of a well digger—states, that he has often stood for hours with his feet in water, without any shirt on, and the water dripping on him from above, without any inconvenience in point of health. About the 6th of April, 1844, being engaged in walling a well, and having his attention called off by some one above him, he struck the ring finger of his left hand a heavy blow with his stone hammer, just upon the root of the nail. From this he experienced a great deal of pain during the day, but did not quit his work. In the evening, observing a quantity of blood collected under the nail, (the blow not having broken the skin or nail,) he went to a smith's shop and with a file made an opening in the nail, through which the blood escaped. Was considerably annoyed by pain in the finger and arm during the following day, after which, however, it appeared to get better very rapidly, and he soon ceased to regard the injury.

On Sunday, April 28th, 22 days after the occurrence of the accident, (his health and occupation having been as usual,) his attention was attracted by a stiffness in his jaws,—not being able to use them with the same facility, or to get a morsel of the usual size between them. There was no pain, but the ordinary relation between volition and execution was slightly disturbed,—the teeth not coming together with the same regularity of interval and effect as is usual in mastication. This difficulty increasing, called to his mind, what otherwise would have escaped notice, the circumstance of his having felt a strange sensation about his heart once or twice during the preceding 24 hours. The stiffness of the jaws continued to augment during the 29th, still unaccompanied by pain. On the 30th felt for the first time a pain in the middle of the breast, and stiffness in the back of the neck, it being now impossible for him to open his jaws to a distance of half an inch.

On the first of May the pain behind the sternum became more severe, and was accompanied by a board-like stiffness in the breast, feeling as though he was compressed between two immovable surfaces. During the day his respiration became implicated, and was irregular, as indicated by irregular jerking speech and misplaced pauses. At this time he began to feel the pain augment whilst speaking. Pain in the chest at times quite severe as evening approached.

Up to the close of the day the affection was confined entirely to the jaws, chest, and back of the neck. During this day he first applied for medical advice, having been well enough to walk several miles on the preceding day. A slight spasmodic twitching of the head was observed; nothing to remark in the finger. Ordered to soak the finger well, and apply a laudanum poultice, and take a pill consisting of half a grain of ext. of belladonna with three grains of the powder of ipecac. and opium, (Dover's Powder,) every three hours. Has had a mustard plaster to his breast, and a ginger poultice to his jaws, without effect.

May 2d, 4 o'clock. Rigidity and spasmodic contraction extended, involving the lower extremities,—the upper remaining free,—head drawn far back without the ability to bend it forward; jaws nearly closed, say quarter of an inch apart; very much distressed by the stabbing pain, and weight in the chest; avoids speaking, as it gives pain and a sensation of something rising in the throat, which almost chokes him until he ceases; considerable twitching in the muscles not affected; a restless stretching out of the limbs, as in yawning, then flexing them again with the same slow steady motion; dull expression of countenance, and eyes half closed; slight aversion to, and difficulty in swallowing, sometimes getting morsels of fluid into his mouth and then ejecting them forcibly, with a hissing sound between his teeth, as if by a sudden strong contraction of the diaphragm; slight hardness and contraction of the muscles along the spine, giving to the body an inclination backward; has perspired pretty freely all day, particularly upon the forehead and chest; pulse regular and tolerably full, say 68; rest during the night pretty good; bowels costive.

Has applied the poultice as directed, but missed the pills once or twice on account of the difficulty in swallowing them. Ordered a rag wet with ol. tereb. to be applied under the poultice, and both to be frequently renewed. Take 30 drops of laudanum every hour, until the spasm abates, or the pain is relieved. Diet of mush and milk, made thin.

May 3d, 10 o'clock. Rested tolerably; cannot walk without support, owing to increasing stiffness in his legs. Muscles on the back and back of the neck rigid and board-like; those of the lower extremities, particularly on the back of the thigh, becoming hardened, but not much contracted; pain in the chest unabated; jaws stationary as at last visit; difficulty in swallowing not increased; distressing difficulty in breathing at times; pulse as at last visit; bowels constipated; skin quite moist, and moderately warm. Ordered round blisters six inches in diameter to the epigastrium, an opium pill (one grain) every two hours, until the pain abates, or a disposition to sleep supervenes; also ten grains of calomel at once.

6 o'clock. Slight abatement of the distressing pain, but the rigidity of the muscles (upper extremities excepted) still increasing; body bending still farther backwards; jaws nearly closed, having been at one time entirely shut, but by frictions with turpentine have become very slightly separated again; has had some spasms, lasting for a few moments, in which the body was gradually bent back to a considerable extent, and then gradually relaxed to its former slightly curved position, each contraction being accompanied by great pain. Directed treatment to be continued.

4th, 9 o'clock. Spasms not so frequent this morning as during the night, when he had several, in one of which he was nearly thrown out of bed, from its having occurred whilst lying upon his back, with pillows under his back and shoulders. Rigidity or tonic contraction increased, insomuch that the trunk and lower extremities are immovable, except when arched backwards by the spasm; has slight strangury, with griping pains in the stomach; nausea; no alvine evacuation; jaws much the same; pulse 96, softer than before; respiration more easy; skin moist; countenance somewhat sunken; slight mental depression.

Applied half grain sulph. of morphia to the blistered surface, and directed one drop of croton oil in pill, every three hours, until it operates, or until three shall have been taken, afterwards continuing the opium as before.

6 o'clock. Little change since last visit; pulse 92; no evacuation of bowels; perspiration free. Opium and poultices continued, the former increased to one pill every hour if the pains increase.

5th, 10 o'clock. Spasms increased in violence, although not quite so frequent. One occurred during the visit, which the attendant says was less severe than many which preceded it. In this the patient was gradually bent backward to an extent of about two-thirds of the circumference of a circle, and then as gradually relaxed to his previous position, the whole exacerbation occupying about 40 seconds, and accompanied with demonstrations of great pain. The segment of a circle formed by the body during the spasm was not regular, the lower extremities not bending at the knee joint, whilst the vertebral column was strongly bent. Rigidity of the muscles between the paroxysms much as before; jaws a little less rigid, allowing a partial view of the tongue, which appeared furred in the centre; pulse 88; respiration again more interfered with by spasmodic action of the diaphragm; expression of countenance anxious; no alvine evacuation. Directed an injection of salt and water; applied half grain sulph. of morphia to the blister, and continue opium, and poultices to the finger.

6½ o'clock. Has had five dejections, consisting of scybalæ; pains in abdomen somewhat relieved; spasms not quite so frequent or extensive; muttering during sleep. Directed the intervals between the opium pills to be increased to four hours, recurring again if the symptoms should increase.

6th, 10 o'clock. Passed an uncomfortable night; troubled by nausea and bilious vomiting, which is very distressing, as nearly all the matter thrown off has to pass through his nose; fears that he shall die of strangulation if it returns; had three evacuations, dark coloured and watery; had pains all through his body in the night, between the spasms; pulse 76; anorexia; mental depression; and complains of pain in the occiput, from resting so much weight upon it when lying upon his back. Opium continued every two hours, with a little milk punch to correct the stomach.

6 o'clock. Much more comfortable, as the stomach is entirely settled; no recurrence of nausea; in other respects much the same; complains somewhat of exhaustion.

7th, 9 o'clock. Passed a very bad night; had several bad spasms towards morning, one of which was much worse than any preceding, and left him in a state of exhaustion, which the attendants mistook for death. According to the directions given for such a contingency, he took 30 drops of laudanum every hour for several hours, which finally seemed to allay the pain and spasm; pulse 92, smaller than usual; skin moist; and some disposition to drowsiness. Applied half grain sulph. of morphia to blister; suspend opium. Diet of ground rice.

5 o'clock. Feels better; countenance brighter; had one stool of a more natural character; spasms not so freequent; in other respects much the same. Applied half grain sulph. of morphia to the blister, still suspending opium.

8th, 8½ o'clock. Feels better, has taken no opium for 17 hours; has numbness and slight stiffness in the arms and hands for the first time; spasms much the same; jaws a little closer than at last visit; pulse 86, rather more full; is restless to some degree, moving without apparent cause. Resume opium every four hours.

5 o'clock. Much as at last visit, except that the skin is more dry, and the stiffness of the arms and hands not so marked.

9th, 9½ o'clock. Had a spasm during the night which seemed to the attendant like a fit; rigidity increased, the muscles feeling as though carved out of wood; pulse 80, and full; skin dry. Continue opium more freely, and diminish the diet.

5½ o'clock. Rigidity stationary; pulse full and hard; epistaxis; also a slight discharge of blood from the rectum; coughed up some clotted blood; spasms diminished in force and frequency; had three thin stools during the day. Venesection ℥x; opium at wider intervals; light diet.

10th, 10 o'clock. Rested tolerably well; no spasms of consequence through the night; rigidity much the same; pulse 82; skin dry; epistaxis. Continue opium at extended intervals; poultices to finger, and light diet.

6 o'clock. Had a spell of coughing, which brought away a quantity of clotted blood, being accompanied by a sense of choking. This was succeeded by severe pain in the chest and neck, for the relief of which he took two doses, of 25 drops each, of laudanum, which has somewhat stupified him for the first time; had two watery stools during the day. Directed one grain of calomel with the opium every four hours, continuing poultices and diet.

11th, 9 o'clock. Feels more comfortable; spasms very rare and slight; rested tolerably well; pulse 90, moderately full; respiration comparatively free; rigidity of muscles much the same; skin rather more moist; some diarrhœa, with tenesmus. Opium to be suspended. Calomel, diet and poultices continued.

6 o'clock. Not so well; complains of feverishness; dryness of mouth and throat, and some pain in the chest; pulse quicker and more frequent; respiration more hurried; wandering pains in the back; rigidity much as before. Opium resumed, calomel and poultices continued, extremities bathed with tepid water, and diet reduced.

12th, 10 o'clock. Feels badly; rested badly; rigidity increased, with slight twitchings; pulse 100, rather small; had two dark watery stools; skin dry; and complains much of dryness of mouth and throat. Continue calomel, opium, &c., using frictions with whiskey and cayenne pepper.

13th, 9 o'clock. Feels much the same; rigidity abated since the frictions; tendency to diarrhœa increasing; pulse 102, small; respiration slightly hurried. Continue treatment.

6 o'clock. Not much change; diarrhœa increasing. Directed an ounce of castor oil. Calomel and opium to be stopped until it has operated.

14th, 2 o'clock. Diarrhœa checked; pulse 90; in other respects much the same. Directed an oval blister 3 by 6, lengthwise to the top of the spinal column, to be dressed with mercurial ointment. Calomel and opium resumed.

15th, 9 o'clock. Complains of debility, and dryness of the mouth and throat. Pulse 110, small; respiration hurried; skin dry; anorexia; rigidity as before. Directed ten drops of oil of turpentine every three hours, in emulsion. Calomel continued, opium diminished.

16th, 9 o'clock. Slept heavily all night, with his eyes half closed—towards morning muttering delirium; pulse 125, small; respiration suspicious, with a long double breath at intervals of a minute or two; had five black watery dejections since last visit; skin moist and clammy; rigidity notably abated; complains of great prostration; disinclination to speak or answer questions, allowing some time to elapse before answering, as though in deep thought; marked hollowness of countenance and emaciation; lies perfectly still, except when touched, when he starts; even the hand laid on the outside of the bed-clothes goes through him like an electric shock, as he expresses it. Continue emulsion and calomel as before, reducing opium to one pill every 6 hours. A little milk punch, with diet.

5½ o'clock. Much better; rigidity abated; wet from head to foot by profuse perspiration, all the bed clothing for many inches around him being saturated with sweat; pulse 135; respiration hurried; diarrhœa checked. Suspend opium until next visit. Continue the remainder.

17th, 9½ o'clock. Much better, and sleeping very quietly; pulse 132; respiration easy and regular; skin very moist; appetite better than for some time; rested tolerably well, but sweats a great deal. Treat by last directions.

6 o'clock. Seems much better; countenance clear; jaws and muscles generally relaxing somewhat; has slight command over his legs; relaxation in neck and back to such an extent as to allow him to assume a half sitting posture; no diarrhœa or pain in the chest since last visit; pulse 130; tongue coated with brown fur; skin quite moist; countenance cheerful. Continue as before, changing the punch for egg nogg, and bathing the extremities in tepid water.

18th, 9½ o'clock. Still continues to get better. Suffers himself to be brought into a sitting posture without pain; can use his lower extremities better; rested comfortably; pulse 132; respiration easy; skin moist; tongue not so much loaded, and better in colour. Continue treatment.

5½ o'clock. Found him propped into a sitting posture, re laxation having taken place to a great extent; pulse 132; skin moist; had two dejections, hard, bloody, and accompanied with tenesmus and prolapsion of the rectum; respiration hurried; every few minutes draws a deep breath or sigh, in which all the respiratory muscles are brought into action; complains much of dryness of the mouth and throat; has lost his memory in great measure, forgetting from one hour to another what transpires. Continue treatment, except the opium.

19th, 9 o'clock. Complains very much of griping pains in his bowels, which were so bad in the night that he took 40 drops of laudanum, from which he obtained relief. Has had three stools this morning, of hard matters, accompanied with some blood and distressing tenesmus; pulse 120; respiration hurried, but more regular; skin moist; tongue covered in the centre by a cinnamon coloured fur; has an abrasion on the sacrum from the use of the bed pan. Continue the turpentine emulsion and diet. 4½ o'clock. Feels better; dejections not so frequent; countenance sunken; pulse 130; muscles still relaxing; can move himself in any direction. One ounce of castor oil, and continue the emulsion.

20th, 12 o'clock. Appears to be improving in every respect; had four dejections during the night, accompanied with little pain. Has been out of bed this morning and sat upon a chair; indicates an almost natural condition of the muscles; can open his jaws to the extent of an inch or more; respiration easy and regular, although frequent; pulse 120; skin quite moist; answers more promptly and remembers better. Directed a small quantity of flaxseed mucilage and lard to be thrown into the rectum, and emulsion and diet continued.

21st, 9 o'clock. Found patient sitting up, on a chair, eating his breakfast, being now able to masticate his food almost as usual. Had two dejections yesterday, and one this morning, the latter being of a dark chocolate colour, about the consistence of gruel, with old clots of blood through it; pulse 122; tongue tolerably clean; skin moist, though drier than usual; respiration regular, and scarcely more frequent than natural; has no pain, except at some of the stools, when he complains slightly. Directed mucilaginous injection, which has been neglected.

At this time the patient passed from under the writer's notice. He has since been assured, however, from the patient himself, through one of his friends, that the recovery progressed to the establishment of perfect health, without further obstruction.

